# Knowledge, attitudes and practices towards rabies: A survey of the general population residing in the Harare Metropolitan Province of Zimbabwe

**DOI:** 10.1371/journal.pone.0246103

**Published:** 2021-01-28

**Authors:** Reverend M. Spargo, Andre Coetzer, Francis T. Makuvadze, Sylvester M. Chikerema, Vaida Chiwerere, Esnath Bhara, Louis H. Nel

**Affiliations:** 1 Division of Epidemiology and Disease Control, Department of Veterinary Services, Harare, Zimbabwe; 2 Department of Biochemistry, Genetics and Microbiology, University of Pretoria, Pretoria, South Africa; 3 Global Alliance for Rabies Control SA NPC, Pretoria, South Africa; 4 Department of Paraclinical Veterinary Studies, University of Zimbabwe, Harare, Zimbabwe; 5 Department of Clinical Veterinary Studies, University of Zimbabwe, Harare, Zimbabwe; University of Lincoln, UNITED KINGDOM

## Abstract

**Background:**

Rabies remains endemic to the Harare Metropolitan Province of Zimbabwe, with a lack of public participations potentially contributing to the limited success in eliminating the disease. We hypothesized that rabies intervention campaigns were less successful than they could be as a result of poor understanding of the disease at the community level, and thus aimed to identify the knowledge, attitudes, and practices towards rabies in the province.

**Methods:**

A cross-sectional survey, using a semi-structured questionnaire, was implemented between January 2017 and June 2018 across the province and data were collected from 798 respondents. Frequency distributions and logistic regressions were undertaken to determine the factors associated with the adequacy of the prevailing rabies knowledge, pet ownership characteristics and the existing preventative practices.

**Results:**

The results of our study suggested that the majority of the respondents (92%) had heard of rabies. However, the level of rabies knowledge could be classified as “adequate” in only 36% of respondents. The multivariate logistic regression analysis indicated that pet ownership and type of occupation were statistically associated with a better understanding and knowledge of the disease. Off all the respondents, 49% owned at least one dog or cat and suburb density and occupation were statistically associated with owning a pet. Amongst the pet owners, 57% consulted an animal health practitioner at least once a year and 75% were aware of a rabies vaccine for their pets. The multivariate logistic regression analysis indicated that age, education and gender were statistically associated with pet owners taking their pet(s) to an animal clinic.

**Conclusion:**

This study showed that the majority of the respondents lacked comprehensive knowledge about rabies, with the knowledge pertaining to health seeking behaviour and the importance of rabies vaccination being the most lacking. Additional public education relying on key messages, aimed at the different target audiences, is required in the province.

## Introduction

Rabies, caused primarily by the *Rabies lyssavirus* (RABV), is a neglected zoonotic disease that is transmitted mainly by domestic dogs (*Canis familiaris*) [1]. Dogs, which account for 99% of all human rabies cases, most significantly affect human populations in developing countries in Asia and Africa [2]. Despite being a vaccine preventable disease in humans and animals [1], the most recent predictive burden models estimate that rabies still kills more than 59,000 people globally every year [2]. In the rabies-endemic country of Zimbabwe, it is estimated that more than 400 people succumb to dog-mediated rabies annually [2]. Although these human rabies cases are mostly limited to the rural areas of the country, rabies was first detected in Zimbabwe’s most densely populated urban province (the Harare Metropolitan Province (HMP)) in 2010 [3]. Since then, it had become endemic and 459 animal rabies cases had been recorded within the province between 2010 and 2019 [4].

In an effort to control and eliminate rabies within the province, both dog vaccination campaigns (reaching between 6% and 12% of the province’s estimated dog population in 2018 and 2019 respectively) and general community awareness activities were implemented by the Ministry of Agriculture [4, 5]. In support of these efforts, a study aimed at gaining an improved understanding of the epidemiology of the disease transmission in the province found that approximately 80% of the rabies-positive samples had been collected from dogs that had been “owned, but unvaccinated” animals. As such, the owners should have, by law, had their companion animals vaccinated against rabies every year [3, 6].

Considering the endemicity of rabies in the HMP and the apparent ignorance regarding the legal requirement and the value of dog vaccination in the prevention of human rabies, it would be prudent to enhance public awareness with regards to rabies transmission, clinical signs, prevention and control. As a first step towards creating specific educational and awareness plans, the aim of this study was to undertake a knowledge, attitude and practice (KAP) survey in the HMP using a cross-sectional questionnaire. The findings of such a survey would enable stakeholders to determine existing knowledge gaps within the population, enabling community-based targeted communication strategies to be developed and implemented in the future.

## Methods

### Ethical considerations

Permission to conduct the research was granted by the Ethics and Animal Welfare Sub-committee of the Department of Veterinary Services (DVS) (Approval number: 009/2018*)*. The Research Ethics Committee (Faculty of Health Sciences, University of Pretoria, South Africa) approved the planned retrospective analysis of the data in the study (Approval number: 68/2019).

### Study area

**Fig 1:**
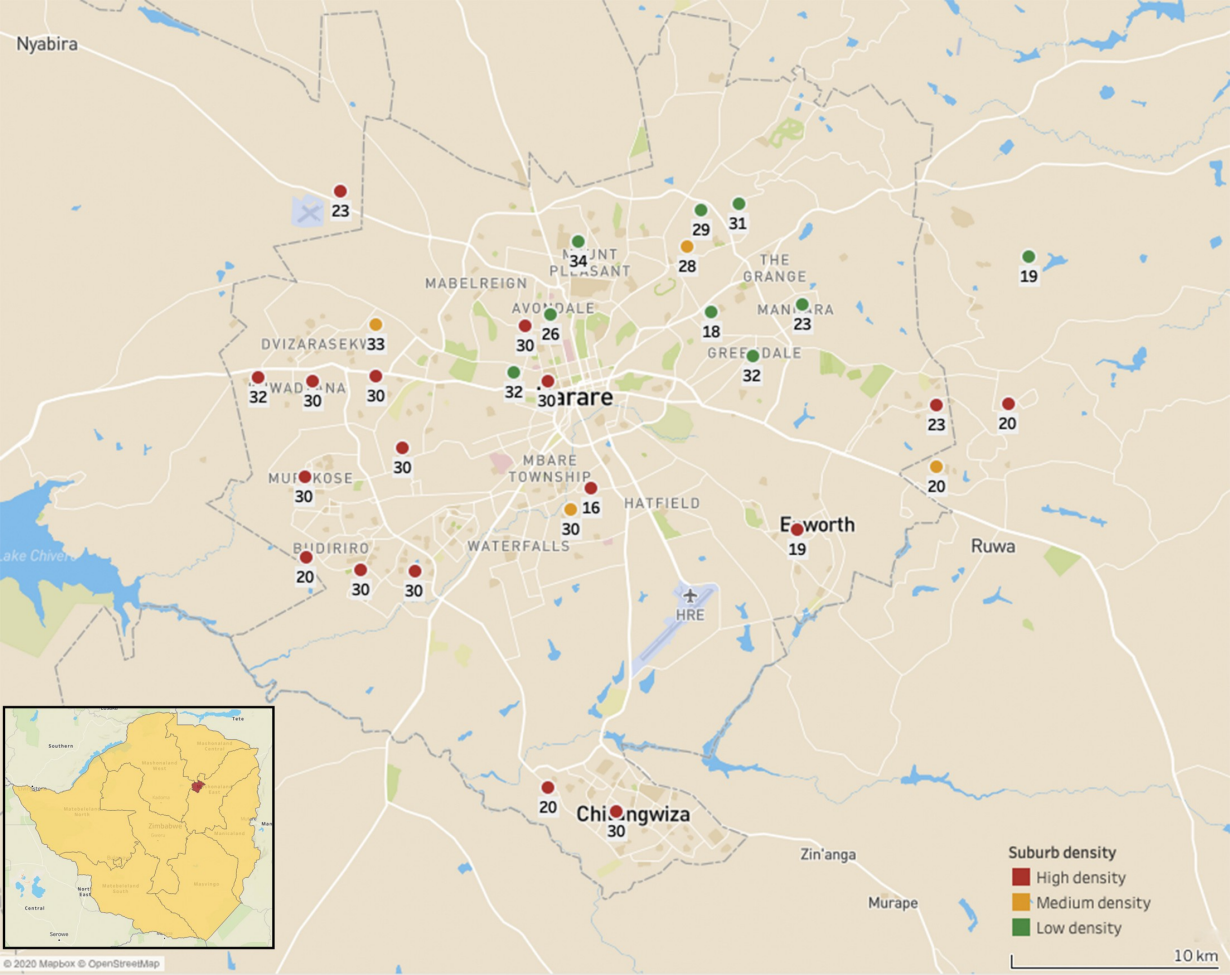
Map of the Harare Metropolitan Province showing locations of sampled suburbs, disaggregated by suburb density. The number associated with each dot represents the number of respondents per suburb. The dots on the maps represent the geographical centre of the suburb and do not represent the size of the suburb in any way. The figures was generated using the Tableau Desktop software package (Version 2020.02) and OpenStreetMap geodata (https://www.openstreetmap.org).

The HMP, situated in the Northeast of Zimbabwe, consists of the capital city of Harare and two other settlements–Chitungwiza and Epworth. Covering approximately 870km^2^, the estimated human population in the HMP is 2,8 million people [7], with approximately 175,000 dogs [8] residing in the province (Fig 1).

### Study design

In an effort to gain a representative panel within the broader catchment area of the HMP, respondents residing in low- (*n = 9*), medium- (*n = 4*) and high-density (*n = 17*) suburbs were included in the investigation (Fig 1). This ensured that respondents from generally high (low density suburbs), moderate (medium density suburbs) and low (high density suburbs) income levels were surveyed [7]. The cross-sectional study, relying on a semi-structured questionnaire (S1 File), was conducted from January 2017 to June 2018 at community gathering points (e.g. shopping centres, community gardens, etc.) and households within each selected suburb in the province.

The pre-testing of the questionnaire was done in three suburbs of the study area, with 15 surveys undertaken during the pre-testing phase. No further alterations were required to the questionnaire and the survey was subsequently implemented across the HMP. During the face to face interviews, the respondents were randomly selected at the various sampling locations before the purpose of the study was explained in detail by the survey facilitator. While no sub-populations were specifically targeted during the simple random study, individuals younger than the age of 16 were included in the study–permitting that their legal guardian also gave consent. With children younger than 15 years accounting for approximately 40% of all human rabies cases in Africa and Asia [9], the inclusion of minors in our study ensured that one of the most at-risk sub-populations was included in the investigation.

Upon commencing the study, adults who gave their verbal consent to participate in the study were interviewed in either English or Shona and the answers were documented. After completing the survey, the respondent signed the completed questionnaire with their name and surname. In the case of minors (individuals younger than 16 years of age), both the legal guardian and minor gave verbal consent for the minor respondent to participate in the study. After the final answers had been captured, both the minor respondent and legal guardian signed the completed questionnaire with their names and surnames. Individuals who did not wish to participate–and thus did not give verbal consent–were omitted from the study and not interviewed. All information obtained from the questionnaire was treated as private and confidential at all times.

Instead of calculating the required sample size before the onset of the study, the survey was implemented across the province and the final sample size was scrutinized in terms of sufficiency. To this end, the confidence interval was determined using a website-based sample size calculator (https://www.surveysystem.com/sscalc.htm). By setting the confidence level at 95% and assuming that 50% (default) of the population would have knowledge and awareness about rabies, the confidence interval was determined to be 3.5. This was deemed to be sufficiently small and no further sampling was required.

### Statistical analysis

Data collected from each questionnaire was captured in excel, filtered, checked for completeness and exported into STATA version 14 (College Corporation Station, TX, USA), after which a descriptive analysis was conducted for the entire dataset.

To determine the “knowledge about rabies” predictor variables, respondents were categorized as having either an “adequate” or “inadequate” knowledge about rabies by using a method described elsewhere [10]. Briefly, respondents were assessed based on their ability to answer five key questions designed to assess their knowledge about rabies, viz. i) identify notable signs in rabid animals, ii) identify a potential reservoir species for rabies, iii) identify at least one mode of rabies transmission, iv) mention the appropriate health seeking behaviour after an exposure has occurred, and v) mention the appropriate course of action to be taken with a suspect rabid animal after someone has come into contact with it. A respondent’s knowledge of rabies was considered adequate if they correctly answered all five questions. Likewise, if a respondent answered any of the five questions incorrectly, or could not answer a question, their rabies knowledge was considered inadequate (S1 Table).

The socio-demographic characteristics were summarised and the adequacy of rabies knowledge, pet ownership characteristics and practices towards rabies control were examined using logistic regression modelling. Firstly, a univariable logistic regression analysis was used to calculate the odds ratios (ORs) and the corresponding 95% confidence intervals (CI) for the various socio-demographic characteristics. Following on from this, a forward stepwise multivariate logistic regression analysis was undertaken as described elsewhere [10]. Briefly, the selection of predictor variables for the multivariate logistic regression analysis was determined using the likelihood ratio test, with only predictor variables that had a p-value of ≤ 0.2 being included in the final model. The multivariate logistic regression analysis was undertaken by adding the significant predictor variables with the smallest p-value first. The remaining significant predictor variables were subsequently added in a stepwise manner and the predictor variables with a p-value of < 0.05 were retained in the model. In order to assess confounding effects, predictor variables that were not selected for the final logistic regression model were added and if the coefficient of the predictor variables changed by more than 25%, the additional variable was deemed to have a confounding effect. No such confounders were, however, detected in this study. Lastly, adjusted OR and their corresponding 95% CIs were derived from the final multivariate logistic regression model.

## Results

### Socio-demographic characteristics

The socio-demographic characteristics of the 798 respondents included in the study are summarized in Table 1. Of all respondents, 49.87% were female and 50.13% were male, with 42% of the respondents falling in the 13–18 years age bracket and 56% of the respondents originating from high density suburbs. Lastly, while various occupations were randomly surveyed, students (69%), teachers (10%) and healthcare professionals (2%) were occupations that appeared most frequently and were thus included in the analyses (Table 1).

**Table 1:** Socio-demographic characteristics of the respondents included in the study.

Characteristic	Frequency *n* (%)
**Gender**	
Female	398 (49.87)
Male	400 (50.13)
**Place of residence**	
Low density suburb	244 (30.58)
Medium density suburb	111 (13.91)
High density suburb	443 (55.51)
**Level of education**	
Primary education	192 (24.06)
High school	338 (42.36)
Certificate	130 (16.29)
Diploma	66 (8.27)
Degree	72 (9.02)
**Occupation**	
Student	553 (69.30)
Teacher	78 (9.77)
Health professional	18 (2.26)
Other	149 (18.67)
**Age**	
<13	192 (24.06)
13–18	342 (42.86
19–24	43 (5.39)
25–30	122 (15.29)
>30	99 (12.41)
**Pet owner**	
Yes	395 (49.50)
No	403 (50.50)

### Knowledge about rabies

**Fig 2:**
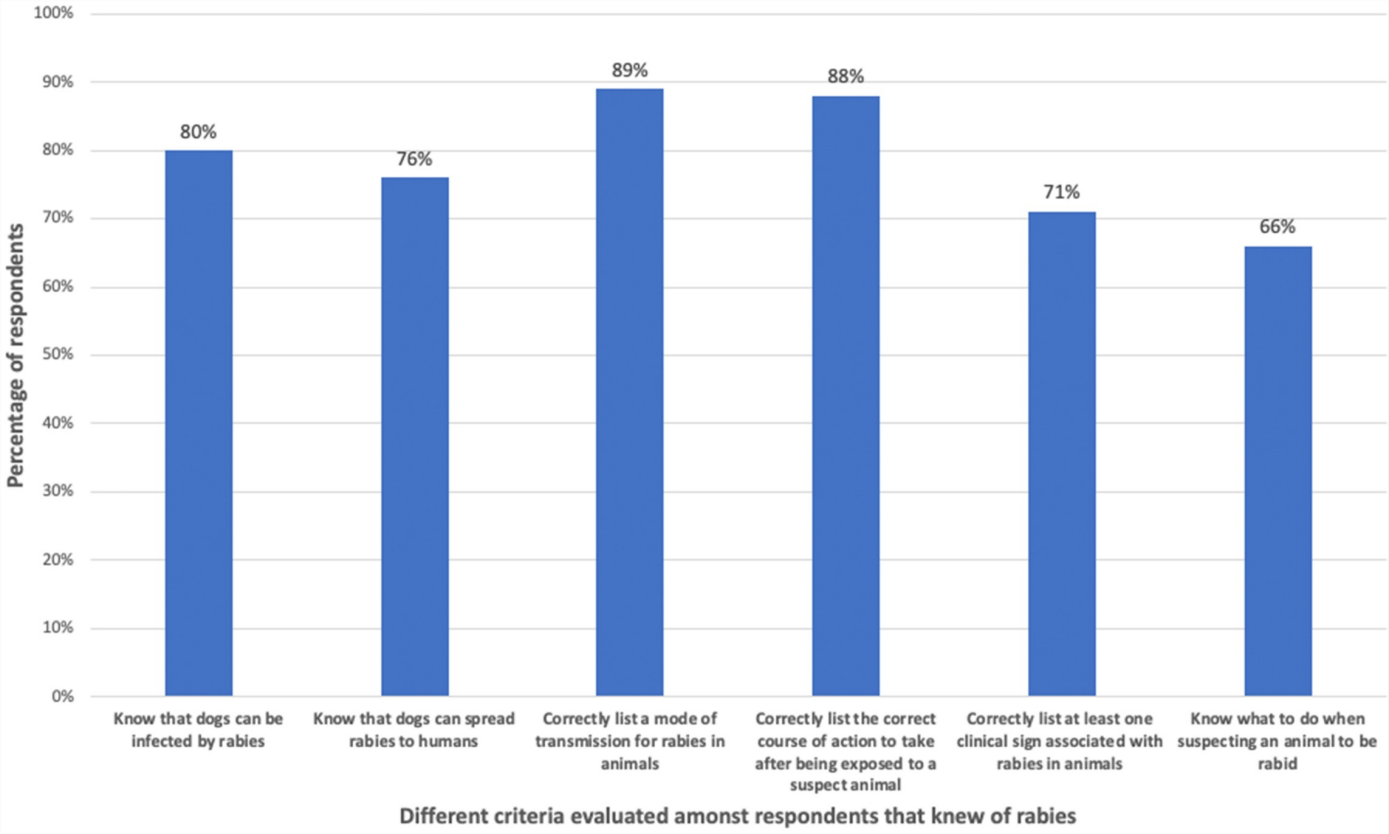
Knowledge of respondents that knew of rabies, disaggregated by different knowledge criteria.

Of all respondents, 92% (735/798) had heard of “rabies”, while 80% of the respondents that knew of rabies also knew that dogs could get infected and 76% knew that dogs could spread the disease to humans. Of the 735 respondents that knew of rabies, 89% (653/735) correctly listed a mode of transmission associated with rabies (e.g. biting or exposure to infected saliva), while 88% (647/735) could list the correct course of action to take after being exposed to a suspect rabid animal (e.g. wound washing, seeking primary healthcare). Furthermore, 71% (524/738) of the respondents that knew of rabies could list at least one clinical sign associated with animal rabies (e.g. salivation, change in behaviour, neurological abnormalities), while 66% (487/735) of the respondents knew what to do when suspecting an animal to be suffering from rabies (e.g. report to the nearest veterinarian, seek help from the local authorities) (Fig 2, Table 2).

**Table 2:** Knowledge of rabies in the study sample.

Characteristic	Frequency *n* (%)
**Number of respondents that had heard of rabies.**	
Yes	735 (92.11)
No	63 (7.89)
**Number of respondents that listed rabies as a disease that could infect dogs.**	
None	166 (20.80)
Rabies	404 (50.63)
Rabies and other disease(s)	186 (23.31)
Other disease(s) (excluding rabies)	42 (5.26)
**Number of respondents that listed rabies as a disease transmitted to humans by dogs.**	
None	235 (29.45)
Rabies	490 (61.40)
Rabies and other disease(s)	68 (8.52)
Other disease(s) (excluding rabies)	5 (0.63)
**Number of respondents that could list the mode(s) of transmission for rabies.**	
None	145 (18.22)
Dog bites	607 (76.26)
Dog bite, licking wounds	46 (5.53)
**Number of respondents that could list the appropriate health seeking behavior after being exposed to a suspect animal.**	
None	53 (6.64)
Wash the wound with antiseptic	89 (11.15)
Wash with wound water	67 (8.40)
Go to the nearest clinic / veterinarian	388 (48.62)
Go to the police	47 (5.89)
Inform the owner	51 (6.39)
A combination of the actions	103 (12.91)
**Number of respondents that could list the clinical signs of rabies in dogs.**	
None	274 (34.34)
Salivation	232 (29.07)
Change in behavior (aggressive)	116 (14.54)
Neurological signs	43 (5.39)
A combination of clinical signs	133 (16.67)
**Number of respondents that could correctly identify the species affected by rabies.**	
None	67 (8.40)
Dog	300 (37.59)
Cat	10 (1.25)
Cat and dog	109 (13.66)
Livestock	12 (1.50)
Dog and livestock	22 (2.76)
Other warm-blooded mammals	5 (0.63)
Dog and other warm-blooded mammals	161 (20.18)
Cat and other warm-blooded mammals	10 (1.25)
All warm-blooded mammals	67 (8.40)
Cat, dog and warm-blooded mammals	35 (4.39)
**Number of respondents that knew the appropriate course of action when encountering a suspect rabid animal.**	
None	76 (9.52)
Report to the nearest veterinarian	280 (35.09)
Report to the police	118 (14.79)
Kill the dog	201 (25.19)
Other	34 (4.26)
A combination of the actions	89 (11.15)
**Source of information on rabies**	
None	235 (29.45)
Media (television and/or radio)	389 (48.75)
Veterinary personnel	54 (6.77)
Clinic/Medical personnel	23 (2.88)
School (primary, secondary or tertiary)	97 (12.16)

Of all respondents, only 36% (286/798) had adequate knowledge about rabies according to our evaluation matrix (S1 Table). The multivariable regression analysis demonstrated that the predictor variables 'pet ownership status’ and ‘occupation’ were both statistically associated with having adequate knowledge about rabies (Table 3). Respondents that owned a pet(s) had a 1.95 greater odds of having adequate knowledge about rabies compared to respondents who did not own pets (aOR: 1.95, 95% CI: 1.44–2.65). Compared to students, healthcare providers had a 9.81 greater odds of having adequate knowledge about rabies (aOR: 9.81, 95% CI: 2.78–34.68), while teachers had a 2.99 greater odds of having adequate knowledge about rabies (aOR: 2.99, 95% CI: 1.81–4.92).

**Table 3:** Univariate and multivariable logistic regression analysis of the association between the socio-demographic variables and “having adequate knowledge about rabies”.

Variable	Categories	Adequate knowledge		Total	OR (95% CI)	P Value	aOR (95% CI)
		Yes	No				
Level of Education	Primary education	51	141	192	Reference		
	High school	114	224	338	1.40 (0.95–2.08)	0.0875	
	Certificate	48	82	130	1.62 (1.00–2.61)	0.0489	
	Diploma	41	25	66	4.53 (2.50–8.19)	< 0.0001	
	Degree	32	40	72	2.21 (1.26–3.89)	0.0058	
Age	<13	51	141	192	Reference		
	13–18	118	224	342	1.46 (0.99 - 2.15)	0.0590	
	19–24	11	32	43	0.95 (0.47–2.02)	0.8950	
	25–30	63	59	122	2.95 (1.83–4.76)	< 0.0001	
	>30	43	56	99	2.12 (1.27–3.54)	0.0038	
Occupation	Student	174	379	553	Reference		Reference
	Teacher	48	30	78	3.49 (2.13–5.69)	< 0.0001	2.99 (1.81–4.92)
	Healthcare professional	15	3	18	10.89 (3.11–38.10)	0.0002	9.81 (2.78–34.68)
	Other	49	100	149	1.07 (0.73–1.57)	0.7409	0.95 (0.64–1.41)
Pet ownership status	No	110	285	395	Reference		Reference
	Yes	176	227	403	2.01 (1.50–2.70)	<0.0001	1.95 (1.44–2.65)
Suburb density	Low	77	167	244	Reference		
	Medium	36	75	111	1.04 (0.64 - 1.68)	0.8697	
	High	173	270	443	1.39 (0.99 - 1.93)	0.0511	
Gender	Female	147	251	398	Reference		
	Male	139	261	400	1.10 (0.82 - 1.47)	0.5200	

OR: Odds Ratio

aOR: Adjusted Odds Ratio

CI: Confidence interval.

In addition to determining whether respondents had an adequate knowledge of rabies, we also endeavoured to gain an improved understanding of which advocacy channels were the most effective within the sampled population. Of all the respondents who knew of rabies (*n = 735*), 23% could list no specific source of information, while 54% got the information from mass media (Television and Radio). Seven percent obtained the information from animal health professionals, while 3% and 13% sourced information about rabies from human health professionals and schools, respectively (Table 2).

### Pet ownership characteristics

Of the 798 respondents, 49% (395/798) were pet owners (e.g. owning a dog or a cat) (Table 2). The multivariable regression analysis demonstrated that the variables ‘occupation’ and ‘suburb density’ were both strongly associated with owning a pet (Table 4). The odds of respondents that resided in high density suburbs owning a pet was 0.59 lower compared to respondents who resided in the low density suburbs (aOR: 0.59, 95% CI: 0.43–0.82). Compared to students, teachers had a 3.12 greater odds of owning a pet (aOR: 3.12, 95% CI: 1.84–5.32), while “other” occupations had a 2.04 greater odds of owning a pet (aOR: 2.04, 95% CI: 1.41–2.96).

**Table 4:** Univariate and multivariable logistic regression analysis of the association between the socio-demographic variables and “owning a pet”.

Variable	Categories	Pet ownership		Total		OR (95% CI)	P value	aOR (95% CI)
		Yes	No					
Level of Education	Primary education	75	117	192	Reference			
	High school	154	184	338	1.31 (0.91 - 1.87)		0.1469	
	Certificate	68	62	130	1.71 (1.09 - 2.68)		0.0193	
	Diploma	46	20	66	3.59 (1.97 - 6.54)		< 0.0001	
	Degree	52	20	72	4.06 (2.24 - 7.33)		< 0.0001	
Age	<13	75	117	192	Reference			
	13–18	154	188	342	1.28 (0.89 - 1.83)		0.1816	
	19–24	22	21	43	1.63 (0.84 - 3.18)		0.1474	
	25–30	76	46	122	2.58 (1.62 - 4.11)		0.0001	
	>30	68	31	99	3.42 (2.05 - 5.72)		< 0.0001	
Occupation	Student	238	315	553	Reference			Reference
	Teacher	55	23	78	3.17 (1.89 - 5.30)		< 0.0001	3.12 (1.84–5.32)
	Healthcare professional	12	6	18	2.65 (0.98 - 7.15)		0.0550	2.68 (0.98–7.29)
	Other	90	59	149	2.02 (1.40 - 2.92)		0.0002	2.04 (1.41–2.96)
Suburb density	Low	141	103	244	Reference			Reference
	Medium	63	48	111	0.96 (0.60 - 1.51)		0.8556	1.07 (0.67–1.69)
	High	191	252	443	0.55 (0.40 - 0.76)		0.0002	0.59 (0.43–0.82)
Gender	Female	211	189	400	Reference			
	Male	184	214	398	1.30 (0.98 - 1.71)		0.0657	

OR: Odds Ratio

aOR: Adjusted Odds Ratio

CI: Confidence interval

### Practices of rabies control

Regarding means of controlling rabies, 61% and 9% of all respondents (*n = 798*) could list dog vaccinations and both prophylactic vaccination in humans as well as dog vaccination, respectively. Furthermore, 51% (410/798) of the respondents were aware of specific legislation requiring the annual vaccination of dogs (Table 5). When investigating the pet owner sub-group specifically (*n = 395*), 62% (245/395) could list dog vaccinations as means to control rabies and 13% (50/395) could list both prophylactic vaccination in humans as well as dog vaccination as a means to control rabies. In addition, 52% (204/395) of the respondents that owned pets were aware of specific legislation pertaining to animal vaccination (Table 5).

**Table 5:** Knowledge of rabies prevention amongst respondents that did not own pets and pet owner.

Characteristic	Respondents that did not own pets (*n = 403*) *n* (%)	Respondents that owned pets (*n = 395*) *n* (%)
**Methods of rabies control**		
None	130 (32.26)	82 (20.76)
Dog vaccination	240 (59.55)	245 (62.03)
Prophylactic vaccination in humans and dog vaccination	23 (5.71)	50 (12.66)
Confinement of pets and elimination of strays	10 (2.48)	18 (4.56)
**Knowledge of pet legislation**		
Yes	206 (51.12)	204 (51.65)
No	197 (48.88)	191 (48.35)
**Taking pet(s) to a veterinary office**		
Yes	---	236 (59.75)
No	---	159 (40.25)
**Regularity of taking pet(s) to a veterinary office**		
Weekly	---	0 (0.00)
Monthly	---	17 (7.20)
Biannual	---	46 (19.49)
Annual	---	164 (69.49)
Unsure	---	9 (3.81)

Of the 395 respondents that were pet owners, 60% (236/395) reported taking their pet(s) to a veterinary office. Within this group, 7% (17/236), 20% (46/236) and 70% (164/236) reported monthly, biannual, and annual veterinary visits respectively. The rest of pet owners who took their pets to a veterinary office (4%) could not recall the frequency (Table 5).

The multivariable regression analysis demonstrated that the variables ‘level of education’, ‘age’ and ‘gender’ were strongly associated with pet owners taking their pet(s) to an animal health professional at least once (Table 6). The odds of males taking their pet(s) to an animal health professional was 1.88 greater compared to females (aOR: 1.88, 95% CI: 1.21–2.91). Compared to respondents with a primary school education, respondents with a high school qualification had a 2.06 greater odds of taking their pet(s) to an animal health professional (aOR: 2.06, 95% CI: 1.16–3.65); respondents with a diploma had a 4.12 greater odds of taking their pet(s) to an animal health professional (aOR: 4.12, 95% CI: 1.86–9.12); and respondents with a degree had a 11.56 greater odds of taking their pet(s) to an animal health professional (aOR: 11.56, 95% CI: 4.36–30.68). Compared to respondents under the age of 13 years, respondents aged 13–18 had a 1.22 greater odds of taking their pet(s) to an animal health professional (aOR: 1.22, 95% CI: 1.07–1.39); respondents aged 25–30 had a 1.48 greater odds of taking their pet(s) to an animal health professional (aOR: 1.48, 95% CI: 1.27–1.72); and respondents that were 30 years and older had a 1.36 greater odds of taking their pet(s) to an animal health professional (aOR: 1.36, 95% CI: 1.16–1.59).

**Table 6:** Univariate and multivariable logistic regression analysis of the association between the socio-demographic variables and “pet owners seeking animal health services”.

Variable	Categories	Pet owner visiting a veterinarian		Total	OR (95% CI)	P Value	aOR (95% CI)
		Yes	No				
Level of Education	Primary education	29	46	75	Reference		Reference
	High school	90	64	154	2.23 (1.27 - 3.92)	0.0053	2.06 (1.16–3.65)
	Certificate	39	29	68	2.13 (1.09 - 4.16)	0.0263	1.94 (0.98–3.82)
	Diploma	32	14	46	3.63 (1.66 - 7.92)	0.0012	4.12 (1.86–9.12)
	Degree	46	6	52	12.16 (4.61 - 32.06)	< 0.0001	11.56 (4.36–30.68)
Age	<13	29	46	75	Reference		Reference
	13–18	90	64	154	1.49 (0.83 - 2.66)	0.1823	1.22 (1.07–1.39)
	19–24	11	11	22	1.59 (0.61 - 4.13)	0.3444	1.12 (0.89–1.40)
	25–30	59	17	76	5.51 (2.70 - 11.22)	< 0.0001	1.48 (1.27–1.72)
	>30	47	21	68	3.55 (1.77 - 7.10)	0.0003	1.36 (1.16–1.59)
Current occupation	Student	238	315	553	Reference		
	Teacher	55	23	78	1.64 (0.90 - 3.00)	0.1109	
	Healthcare professional	12	6	18	23.38 (1.37 - 399.42)	0.02595	
	Other	90	59	149	2.57 (1.51 - 4.38)	0.0005	
Gender	Female	96	88	184	Reference		Reference
	Male	140	71	211	1.80 (1.20 - 2.71)	0.0043	1.88 (1.21–2.91)
Suburb density	Low	87	54	141	Reference		
	Medium	36	27	63	0.83 (0.45 - 1.51)	0.5389	
	High	113	78	191	0.90 (0.58 - 1.40)	0.6403	

OR: Odds ratio

aOR: Adjusted odds ratio

CI: Confidence interval

## Discussion

The prevention and control of rabies should be of the utmost importance, with evidence provided elsewhere showcasing successful control and even the feasibility of elimination [1, 11, 12]. Achieving rabies elimination does, however, not only rely on vaccinating a significant proportion of the at-risk dog population, but on other complimentary activities as well [13]. Amongst those activities, “enhanced education and awareness” is considered one of the crucial elements [13]. In support of this, studies from India, Ethiopia and Tanzania among others, bear testimony to the direct correlation between enhanced public knowledge, a change in attitudes and practices, and the ultimate decrease of rabies burden in affected areas [14–16].

In order to gain an improved understanding of the knowledge of rabies and its control within the HMP, we undertook a province-wide KAP survey focusing on rabies specifically. This was, to the best of our knowledge, the first rabies-specific KAP survey conducted in Zimbabwe and has provided valuable insight in terms of the prevailing rabies knowledge, pet ownership characteristics and practices towards rabies control amongst respondents residing in the HMP.

The result of our study suggested that only 36% of the 798 respondents included in our study had an adequate knowledge of rabies, which was similar to what was observed in Uganda (41%) [17] but lower than findings reported from elsewhere in Africa, *viz*. Nigeria (82%) [18], Ethiopia (56%) [19], Rwanda (56%) [20], Kenya (67%) [21], and Tanzania (96%) [16]. Our findings suggested that the lack of adequate knowledge could be attributed to most of the participants having a good general knowledge of rabies, but lacking the comprehensive knowledge required to be truly knowledgeable about the disease and its prevention or control. For example, the majority of the respondents knew that dogs could get rabies and that dogs transmitted rabies to humans, but very few could adequately answer questions pertaining wound treatment and post-exposure prophylaxis (PEP). In evidence of the fact, our survey suggested that approximately 20% of the respondents mentioned wound washing as a component of wound treatment and only 9% of the respondents were able to list PEP for humans as a way to prevent rabies. While worrisome, these observations were not unique to our study. Similar findings were observed elsewhere on the African continent where several studies also found that the lack of knowledge of post exposure treatment was one of the most significant deficiencies in the knowledge profile of respondents [15–18, 20, 22, 23].

Furthermore, our findings suggested that only the predictor variables ‘Occupation’ and “Pet ownership status’ were strongly associated with having an adequate knowledge of rabies. These observations were similar to those observed in Ethiopia and Nigeria [18, 19], but were also in contradiction to those observed in Rwanda where pet ownership and occupation–along with sex and location–were not strongly associated with rabies knowledge [20].

Considering advocacy channels amongst the respondents that knew of rabies, media sources such as TV and radio were the most used methods (54%), while advice from skilled animal and human health professionals were much lower (7% and 3% respectively). These observations were similar to those observed in India, Rwanda and Uganda where it was reported that mass media was one of the main source of rabies information [17, 20, 24], but were also in contradiction to those observed in Bangladesh where physicians and governmental representatives were the major sources of rabies information [25]. Indeed, medical personnel and veterinarians should, in principle, play a significant role in the dissemination of rabies information, whereas our study suggested that it was not the case in the HMP. In addition, teachers also need to be utilized more effectively. Despite being three times more likely to have adequate knowledge compared to school children, only 13% of the respondents in our study that knew of rabies considered teachers a source of rabies knowledge. A similar observation was found in Ethiopia where only 11% of the respondents considered teachers a source of rabies knowledge–illustrating the need to empower teachers so that they can adequate educate children who are in their formative years. To this end, teachers and tertiary educators would need to be provided with appropriate lesson plans. In evidence of the effectiveness of this approach, the government of the Philippines had incorporated rabies awareness into their official school curriculum–with the life-saving information reaching an estimated 24 million children every year [26].

In light of the strong association between pet ownership and rabies knowledge observed in our study, we endeavoured to gain a better understanding of pet owner characteristics within the HMP. Our results showed that 49% of the respondents within the HMP were pet owners (owning a cat, a dog or a combination of the two). This level of pet ownership was similar to what was observed in other studies in Africa, where pet ownership was reportedly between 40% and 58% [17–19]. Within the pet owner sub-population specifically, 60% of the respondents within the HMP reported taking their pet(s) to a veterinary office. Ninety-six percent of those respondents visited an animal health professional at least once a year, while 75% could list either dog vaccination or dog vaccination and human PEP as a means to control rabies. While respondents in our study were never specifically asked whether they had their pets vaccinated at any point in time, these observations would suggest that approximately 43% of the owned dog population should theoretically have received their annual vaccination against rabies every year. Assuming this to be accurate, the results would be similar to findings from Uganda [17] and Kenya [21] where a low percentage of respondents (between 35% and 43%) reported having their pets vaccinated against rabies, and in contradiction to findings from Nigeria [18], Ethiopia [19] and Rwanda [20] where a much higher percentage of respondents (between 74% and 94%) reported having their pets vaccinated against rabies. Furthermore, this speculative vaccination coverage in the HMP (30%) is well below the recommended vaccination coverage of 70% [1], which could explain the persistence of the disease in the province.

As could be expected from a non-longitudinal observational study, our study did have some limitations. Since the official declaration of the rabies outbreak in 2016, educational and awareness campaigns had been coupled with disease intervention activities across the province. With these educational activities striving to provide people with the basic knowledge about rabies, they could have resulted in a bias in the information presented here–especially in terms of knowledge pertaining to the role that dog vaccination plays in preventing rabies. Furthermore, despite relying on randomly selected participants, the study inadvertently disproportionately sampled specific sub-populations (e.g. teachers and students), which could have influenced the findings of the study. Lastly, the responses could have been biased by the open-ended nature of some of the questions (questions that allowed respondents to give a free-form answer), with the investigators subconsciously guiding the respondent’s answers. Nevertheless, the results presented here did provide valuable insight into the prevailing knowledge, attitudes and practices amongst people living in the HMP.

## Conclusion

This study demonstrated that most people residing in the HMP had a basic general knowledge of rabies. However, an overall lack of comprehensive knowledge was noted in most of the population. By developing and implementing a well-structured Information, Education and Communication (IEC) plan for the HMP–using new and existing advocacy channels to target both the general population and pet owners with specific messages like “vaccinate your pet(s) against rabies”, “immediately wash any animal bites and scratches with soap and running water”, and “get rabies vaccination at your nearest healthcare facility if you have been exposed to an animal bite or scratch”–rabies control activities could become more effective and far reaching, in-turn preventing the needless loss of human and animal lives across the province and beyond.

## Supporting information

S1 Checklist Strobe checklist. (PDF)

S1 File Questionnaire used during the KAP survey of the general population residing in the Harare Metropolitan Province. (PDF)

S1 Table Results of the evaluation matrix that assessed respondent’s rabies knowledge. (PDF)
