# Supplementary material for: Knowledge, attitudes and practices towards rabies: A survey of the general population residing in the Harare Metropolitan Province of Zimbabwe
Source: PLoS One. 2021 Jan 28;16(1):e0246103. doi: 10.1371/journal.pone.0246103 (PMC7842990; doi:10.1371/journal.pone.0246103)
Supplement: S1 File — (PDF) [file pone.0246103.s002.pdf]

Questionnaire 1: KAP survey of general population residing in the Harare Metropolitan Province

Suburb ……………………………………………………………………..

1. Personal details

1. Gender

☐ Female ☐ Male

2. Highest education obtained:

☐ Not completed high school ☐ High school

☐ Certificate ☐ Diploma

☐ Degree

3. Occupation

☐ Student ☐ Teacher

☐ Health professional ☐ Other

4. Age

☐ Below 13 ☐ 13 -18 years

☐ 19- 24 years ☐ 25-30 years

☐ Above 30 years

5. Do you own a pet or pets?

☐ Yes ☐ No

6. If yes, list them

2. Disease information

7. Do you know any diseases that may affect dogs?

☐ Yes ☐ No

8. List some of the diseases that affect dogs

9. Do you know any diseases that can be transmitted from dogs to humans?

☐ Yes ☐ No

10. List some of the diseases that can be transmitted from dogs to human beings

11. Have you ever heard of rabies?

☐ Yes ☐ No

If yes, by whom?

12. Have there been reported rabies cases in your area?

13. How is Rabies transmitted?

14. List some of the animals that can be infected by rabies

15. Do you know the clinical signs of rabies?

☐ Yes ☐ No

16. If yes, list them

17. Do you know anyone who has been bitten by a dog?

☐ Yes ☐ No

18. What steps do you take when someone is bitten by a dog?

19. What do you do to the dog suspected of having rabies?

20. Have you ever visited a veterinarian?

☐ Yes ☐ No

21. Do you know methods for controlling rabies?

☐ Yes ☐ No

If yes, list them

22. How often do you take your pet(s) for vaccination?

23. Do you know any laws(by-laws) concerning the keeping of dogs
