# Supplementary material for: Knowledge, attitudes and practices towards rabies: A survey of the general population residing in the Harare Metropolitan Province of Zimbabwe
Source: PLoS One. 2021 Jan 28;16(1):e0246103. doi: 10.1371/journal.pone.0246103 (PMC7842990; doi:10.1371/journal.pone.0246103)
Supplement: S1 Table — (PDF) [file pone.0246103.s003.pdf]

Respondent	Modes of rabies transmission	Species affected by rabies	Examples of clinical signs	action taken after a dog bite	Action taken when suspecting rabies	FINAL outcome
1	FAIL	FAIL	FAIL	PASS	FAIL	Inadequate
2	PASS	PASS	FAIL	PASS	FAIL	Inadequate
3	PASS	PASS	PASS	PASS	FAIL	Inadequate
4	PASS	PASS	PASS	PASS	FAIL	Inadequate
5	PASS	PASS	FAIL	PASS	FAIL	Inadequate
6	PASS	PASS	PASS	PASS	FAIL	Inadequate
7	PASS	PASS	PASS	PASS	FAIL	Inadequate
8	FAIL	PASS	PASS	PASS	PASS	Inadequate
9	PASS	PASS	PASS	PASS	PASS	Adequate
10	PASS	PASS	PASS	PASS	PASS	Adequate
11	PASS	FAIL	FAIL	PASS	PASS	Inadequate
12	PASS	PASS	PASS	PASS	FAIL	Inadequate
13	PASS	PASS	PASS	PASS	FAIL	Inadequate
14	PASS	PASS	PASS	PASS	FAIL	Inadequate
15	PASS	PASS	PASS	PASS	FAIL	Inadequate
16	PASS	PASS	FAIL	FAIL	FAIL	Inadequate
17	PASS	PASS	PASS	PASS	PASS	Adequate
18	PASS	PASS	FAIL	PASS	FAIL	Inadequate
19	PASS	PASS	FAIL	PASS	PASS	Inadequate
20	FAIL	PASS	FAIL	PASS	FAIL	Inadequate
21	PASS	FAIL	PASS	PASS	FAIL	Inadequate
22	PASS	PASS	PASS	PASS	FAIL	Inadequate
23	PASS	PASS	PASS	PASS	FAIL	Inadequate
24	PASS	PASS	FAIL	FAIL	FAIL	Inadequate
25	PASS	PASS	FAIL	PASS	FAIL	Inadequate
26	PASS	PASS	PASS	PASS	PASS	Adequate
27	PASS	PASS	PASS	PASS	FAIL	Inadequate
28	PASS	PASS	FAIL	PASS	PASS	Inadequate
29	PASS	PASS	PASS	PASS	FAIL	Inadequate
30	PASS	PASS	PASS	PASS	FAIL	Inadequate
31	PASS	PASS	PASS	PASS	PASS	Adequate
32	PASS	PASS	PASS	PASS	PASS	Adequate
33	PASS	PASS	PASS	PASS	PASS	Adequate
34	PASS	PASS	PASS	PASS	PASS	Adequate
35	PASS	PASS	PASS	PASS	PASS	Adequate
36	PASS	PASS	PASS	PASS	FAIL	Inadequate
37	PASS	PASS	PASS	PASS	PASS	Adequate
38	PASS	PASS	FAIL	PASS	FAIL	Inadequate
39	PASS	PASS	PASS	PASS	PASS	Adequate
40	PASS	PASS	PASS	FAIL	FAIL	Inadequate

41	PASS	PASS	PASS	PASS	FAIL	Inadequate
42	PASS	PASS	FAIL	PASS	FAIL	Inadequate
43	PASS	PASS	FAIL	PASS	FAIL	Inadequate
44	PASS	PASS	FAIL	PASS	PASS	Inadequate
45	PASS	PASS	PASS	PASS	PASS	Adequate
46	PASS	PASS	FAIL	FAIL	FAIL	Inadequate
47	FAIL	PASS	FAIL	PASS	PASS	Inadequate
48	PASS	PASS	FAIL	PASS	FAIL	Inadequate
49	PASS	PASS	FAIL	PASS	FAIL	Inadequate
50	PASS	PASS	PASS	PASS	PASS	Adequate
51	PASS	PASS	FAIL	PASS	FAIL	Inadequate
52	PASS	PASS	FAIL	PASS	FAIL	Inadequate
53	PASS	PASS	PASS	PASS	PASS	Adequate
54	PASS	PASS	PASS	PASS	FAIL	Inadequate
55	PASS	PASS	FAIL	FAIL	FAIL	Inadequate
56	PASS	PASS	PASS	PASS	FAIL	Inadequate
57	PASS	PASS	PASS	PASS	PASS	Adequate
58	FAIL	PASS	FAIL	FAIL	FAIL	Inadequate
59	PASS	PASS	FAIL	PASS	FAIL	Inadequate
60	FAIL	FAIL	FAIL	FAIL	FAIL	Inadequate
61	PASS	PASS	PASS	PASS	FAIL	Inadequate
62	PASS	PASS	PASS	PASS	FAIL	Inadequate
63	PASS	PASS	PASS	PASS	PASS	Adequate
64	PASS	PASS	PASS	PASS	PASS	Adequate
65	PASS	PASS	PASS	PASS	PASS	Adequate
66	PASS	PASS	PASS	PASS	PASS	Adequate
67	PASS	PASS	PASS	PASS	FAIL	Inadequate
68	PASS	PASS	PASS	PASS	PASS	Adequate
69	FAIL	PASS	FAIL	PASS	FAIL	Inadequate
70	PASS	PASS	PASS	PASS	PASS	Adequate
71	FAIL	FAIL	FAIL	PASS	FAIL	Inadequate
72	PASS	FAIL	PASS	PASS	PASS	Inadequate
73	PASS	PASS	PASS	PASS	PASS	Adequate
74	PASS	PASS	PASS	PASS	PASS	Adequate
75	PASS	PASS	PASS	PASS	PASS	Adequate
76	PASS	PASS	PASS	PASS	PASS	Adequate
77	PASS	PASS	PASS	PASS	PASS	Adequate
78	PASS	PASS	PASS	PASS	PASS	Adequate
79	PASS	PASS	PASS	PASS	FAIL	Inadequate
80	PASS	FAIL	FAIL	PASS	FAIL	Inadequate
81	PASS	PASS	FAIL	PASS	PASS	Inadequate

82	FAIL	FAIL	FAIL	PASS	FAIL	Inadequate
83	FAIL	PASS	FAIL	PASS	FAIL	Inadequate
84	PASS	PASS	FAIL	FAIL	FAIL	Inadequate
85	PASS	PASS	FAIL	PASS	PASS	Inadequate
86	FAIL	FAIL	FAIL	FAIL	FAIL	Inadequate
87	PASS	PASS	FAIL	PASS	FAIL	Inadequate
88	PASS	PASS	FAIL	PASS	PASS	Inadequate
89	PASS	PASS	PASS	PASS	PASS	Adequate
90	PASS	PASS	FAIL	FAIL	FAIL	Inadequate
91	PASS	PASS	PASS	PASS	FAIL	Inadequate
92	PASS	PASS	PASS	PASS	FAIL	Inadequate
93	PASS	PASS	PASS	PASS	FAIL	Inadequate
94	PASS	PASS	PASS	PASS	PASS	Adequate
95	FAIL	FAIL	FAIL	PASS	FAIL	Inadequate
96	PASS	PASS	PASS	FAIL	PASS	Inadequate
97	PASS	PASS	PASS	PASS	PASS	Adequate
98	FAIL	PASS	PASS	PASS	PASS	Inadequate
99	PASS	PASS	PASS	PASS	PASS	Adequate
100	PASS	PASS	FAIL	PASS	FAIL	Inadequate
101	PASS	PASS	FAIL	FAIL	FAIL	Inadequate
102	PASS	PASS	FAIL	PASS	PASS	Inadequate
103	PASS	PASS	PASS	PASS	PASS	Adequate
104	PASS	PASS	FAIL	PASS	FAIL	Inadequate
105	PASS	PASS	PASS	PASS	PASS	Adequate
106	FAIL	PASS	FAIL	PASS	PASS	Inadequate
107	PASS	FAIL	FAIL	PASS	FAIL	Inadequate
108	PASS	PASS	PASS	PASS	PASS	Adequate
109	PASS	PASS	PASS	PASS	PASS	Adequate
110	PASS	PASS	PASS	PASS	PASS	Adequate
111	PASS	PASS	FAIL	PASS	FAIL	Inadequate
112	PASS	PASS	PASS	PASS	PASS	Adequate
113	PASS	PASS	FAIL	PASS	PASS	Inadequate
114	FAIL	PASS	FAIL	PASS	PASS	Inadequate
115	PASS	PASS	PASS	PASS	PASS	Adequate
116	PASS	PASS	PASS	FAIL	PASS	Inadequate
117	PASS	PASS	FAIL	PASS	FAIL	Inadequate
118	PASS	PASS	FAIL	PASS	PASS	Inadequate
119	PASS	PASS	PASS	FAIL	FAIL	Inadequate
120	PASS	PASS	PASS	FAIL	FAIL	Inadequate
121	PASS	PASS	PASS	FAIL	FAIL	Inadequate
122	PASS	PASS	PASS	FAIL	FAIL	Inadequate

123	PASS	PASS	PASS	FAIL	FAIL	Inadequate
124	PASS	PASS	PASS	FAIL	FAIL	Inadequate
125	PASS	PASS	PASS	FAIL	FAIL	Inadequate
126	PASS	PASS	PASS	FAIL	FAIL	Inadequate
127	PASS	PASS	PASS	FAIL	FAIL	Inadequate
128	PASS	PASS	PASS	PASS	PASS	Adequate
129	PASS	PASS	PASS	PASS	PASS	Adequate
130	PASS	PASS	PASS	PASS	PASS	Adequate
131	PASS	PASS	PASS	PASS	PASS	Adequate
132	PASS	PASS	PASS	PASS	PASS	Adequate
133	PASS	PASS	PASS	PASS	PASS	Adequate
134	PASS	PASS	PASS	PASS	PASS	Adequate
135	PASS	PASS	PASS	PASS	PASS	Adequate
136	PASS	PASS	PASS	PASS	PASS	Adequate
137	PASS	PASS	PASS	PASS	PASS	Adequate
138	PASS	PASS	PASS	PASS	PASS	Adequate
139	PASS	PASS	PASS	PASS	PASS	Adequate
140	PASS	PASS	PASS	PASS	PASS	Adequate
141	PASS	PASS	PASS	PASS	PASS	Adequate
142	PASS	PASS	PASS	PASS	PASS	Adequate
143	PASS	PASS	PASS	PASS	PASS	Adequate
144	PASS	PASS	PASS	PASS	PASS	Adequate
145	PASS	PASS	PASS	PASS	PASS	Adequate
146	PASS	PASS	PASS	PASS	PASS	Adequate
147	PASS	PASS	PASS	PASS	PASS	Adequate
148	PASS	PASS	PASS	PASS	FAIL	Inadequate
149	FAIL	PASS	PASS	PASS	FAIL	Inadequate
150	FAIL	PASS	PASS	PASS	FAIL	Inadequate
151	FAIL	PASS	PASS	PASS	FAIL	Inadequate
152	PASS	PASS	PASS	FAIL	PASS	Inadequate
153	PASS	PASS	FAIL	PASS	FAIL	Inadequate
154	PASS	PASS	FAIL	PASS	FAIL	Inadequate
155	PASS	PASS	FAIL	PASS	PASS	Inadequate
156	PASS	PASS	PASS	PASS	PASS	Adequate
157	FAIL	FAIL	FAIL	FAIL	PASS	Inadequate
158	PASS	PASS	PASS	PASS	PASS	Adequate
159	PASS	PASS	PASS	FAIL	PASS	Inadequate
160	FAIL	PASS	FAIL	PASS	FAIL	Inadequate
161	FAIL	PASS	FAIL	FAIL	FAIL	Inadequate
162	PASS	PASS	PASS	PASS	PASS	Adequate
163	FAIL	PASS	PASS	FAIL	FAIL	Inadequate

164	PASS	PASS	PASS	PASS	PASS	Adequate
165	PASS	PASS	PASS	PASS	PASS	Adequate
166	PASS	PASS	FAIL	PASS	PASS	Inadequate
167	PASS	PASS	FAIL	PASS	PASS	Inadequate
168	PASS	PASS	FAIL	FAIL	FAIL	Inadequate
169	PASS	PASS	PASS	PASS	PASS	Adequate
170	PASS	PASS	PASS	PASS	PASS	Adequate
171	FAIL	PASS	FAIL	FAIL	PASS	Inadequate
172	FAIL	PASS	FAIL	PASS	FAIL	Inadequate
173	PASS	PASS	PASS	PASS	PASS	Adequate
174	PASS	PASS	PASS	FAIL	FAIL	Inadequate
175	PASS	PASS	PASS	FAIL	PASS	Inadequate
176	PASS	PASS	PASS	FAIL	FAIL	Inadequate
177	PASS	PASS	FAIL	FAIL	FAIL	Inadequate
178	PASS	PASS	PASS	PASS	PASS	Adequate
179	FAIL	PASS	FAIL	FAIL	PASS	Inadequate
180	PASS	PASS	PASS	FAIL	PASS	Inadequate
181	PASS	PASS	FAIL	FAIL	PASS	Inadequate
182	PASS	PASS	PASS	PASS	PASS	Adequate
183	PASS	PASS	PASS	PASS	PASS	Adequate
184	PASS	PASS	FAIL	PASS	PASS	Inadequate
185	PASS	PASS	PASS	PASS	PASS	Adequate
186	PASS	PASS	FAIL	PASS	PASS	Inadequate
187	PASS	PASS	PASS	PASS	PASS	Adequate
188	FAIL	FAIL	FAIL	PASS	FAIL	Inadequate
189	FAIL	FAIL	FAIL	PASS	PASS	Inadequate
190	PASS	PASS	PASS	PASS	FAIL	Inadequate
191	PASS	PASS	PASS	PASS	PASS	Adequate
192	PASS	PASS	PASS	PASS	PASS	Adequate
193	FAIL	FAIL	FAIL	PASS	PASS	Inadequate
194	PASS	PASS	PASS	PASS	PASS	Adequate
195	PASS	PASS	PASS	PASS	FAIL	Inadequate
196	PASS	PASS	PASS	PASS	PASS	Adequate
197	PASS	PASS	PASS	PASS	PASS	Adequate
198	FAIL	FAIL	FAIL	PASS	FAIL	Inadequate
199	PASS	PASS	PASS	PASS	PASS	Adequate
200	PASS	PASS	PASS	PASS	PASS	Adequate
201	PASS	PASS	FAIL	PASS	PASS	Inadequate
202	FAIL	FAIL	FAIL	PASS	PASS	Inadequate
203	PASS	PASS	PASS	PASS	PASS	Adequate
204	FAIL	FAIL	FAIL	PASS	PASS	Inadequate

205	PASS	PASS	PASS	PASS	PASS	Adequate
206	FAIL	FAIL	FAIL	PASS	FAIL	Inadequate
207	PASS	PASS	PASS	PASS	PASS	Adequate
208	PASS	FAIL	PASS	PASS	FAIL	Inadequate
209	PASS	FAIL	PASS	PASS	PASS	Inadequate
210	FAIL	FAIL	FAIL	PASS	PASS	Inadequate
211	FAIL	FAIL	PASS	PASS	FAIL	Inadequate
212	PASS	PASS	FAIL	PASS	FAIL	Inadequate
213	FAIL	PASS	PASS	FAIL	FAIL	Inadequate
214	PASS	PASS	PASS	FAIL	PASS	Inadequate
215	FAIL	PASS	PASS	PASS	FAIL	Inadequate
216	PASS	PASS	FAIL	FAIL	FAIL	Inadequate
217	FAIL	PASS	PASS	FAIL	PASS	Inadequate
218	PASS	PASS	PASS	PASS	FAIL	Inadequate
219	PASS	PASS	PASS	PASS	PASS	Adequate
220	PASS	PASS	PASS	FAIL	PASS	Inadequate
221	PASS	PASS	PASS	PASS	PASS	Adequate
222	FAIL	FAIL	FAIL	PASS	FAIL	Inadequate
223	PASS	PASS	FAIL	PASS	FAIL	Inadequate
224	PASS	FAIL	FAIL	PASS	PASS	Inadequate
225	PASS	PASS	PASS	PASS	FAIL	Inadequate
226	FAIL	PASS	PASS	PASS	FAIL	Inadequate
227	PASS	PASS	PASS	PASS	FAIL	Inadequate
228	PASS	PASS	PASS	PASS	FAIL	Inadequate
229	FAIL	FAIL	FAIL	PASS	FAIL	Inadequate
230	FAIL	FAIL	FAIL	PASS	FAIL	Inadequate
231	FAIL	FAIL	PASS	PASS	FAIL	Inadequate
232	PASS	FAIL	PASS	PASS	PASS	Inadequate
233	PASS	PASS	PASS	FAIL	PASS	Inadequate
234	FAIL	FAIL	FAIL	PASS	FAIL	Inadequate
235	FAIL	FAIL	PASS	PASS	PASS	Inadequate
236	PASS	PASS	PASS	FAIL	PASS	Inadequate
237	PASS	PASS	PASS	FAIL	PASS	Inadequate
238	PASS	PASS	FAIL	PASS	PASS	Inadequate
239	PASS	PASS	PASS	PASS	FAIL	Inadequate
240	PASS	PASS	PASS	FAIL	PASS	Inadequate
241	PASS	PASS	PASS	PASS	FAIL	Inadequate
242	PASS	PASS	PASS	PASS	PASS	Adequate
243	PASS	PASS	PASS	FAIL	PASS	Inadequate
244	PASS	PASS	PASS	PASS	PASS	Adequate
245	PASS	PASS	PASS	FAIL	FAIL	Inadequate

246	PASS	PASS	FAIL	PASS	PASS	Inadequate
247	PASS	PASS	PASS	PASS	PASS	Adequate
248	PASS	PASS	PASS	FAIL	PASS	Inadequate
249	PASS	PASS	PASS	FAIL	PASS	Inadequate
250	PASS	PASS	PASS	PASS	PASS	Adequate
251	PASS	PASS	PASS	PASS	PASS	Adequate
252	PASS	FAIL	PASS	PASS	PASS	Inadequate
253	PASS	PASS	PASS	PASS	PASS	Adequate
254	PASS	PASS	FAIL	PASS	FAIL	Inadequate
255	PASS	PASS	PASS	PASS	PASS	Adequate
256	PASS	PASS	PASS	FAIL	PASS	Inadequate
257	PASS	PASS	PASS	FAIL	PASS	Inadequate
258	PASS	PASS	PASS	PASS	PASS	Adequate
259	PASS	PASS	PASS	PASS	PASS	Adequate
260	PASS	PASS	PASS	PASS	PASS	Adequate
261	PASS	PASS	PASS	PASS	PASS	Adequate
262	PASS	PASS	PASS	PASS	PASS	Adequate
263	PASS	PASS	PASS	PASS	PASS	Adequate
264	PASS	PASS	PASS	PASS	FAIL	Inadequate
265	PASS	PASS	PASS	PASS	PASS	Adequate
266	PASS	PASS	PASS	PASS	PASS	Adequate
267	PASS	PASS	PASS	PASS	FAIL	Inadequate
268	FAIL	PASS	PASS	PASS	PASS	Inadequate
269	PASS	PASS	PASS	PASS	PASS	Adequate
270	PASS	PASS	PASS	PASS	FAIL	Inadequate
271	PASS	PASS	FAIL	PASS	PASS	Inadequate
272	PASS	PASS	PASS	PASS	PASS	Adequate
273	PASS	PASS	PASS	FAIL	PASS	Inadequate
274	PASS	PASS	PASS	PASS	PASS	Adequate
275	PASS	PASS	PASS	PASS	PASS	Adequate
276	PASS	PASS	PASS	PASS	PASS	Adequate
277	PASS	PASS	PASS	PASS	PASS	Adequate
278	FAIL	PASS	PASS	FAIL	PASS	Inadequate
279	PASS	PASS	PASS	PASS	PASS	Adequate
280	PASS	PASS	FAIL	PASS	PASS	Inadequate
281	FAIL	PASS	PASS	PASS	FAIL	Inadequate
282	FAIL	PASS	PASS	FAIL	FAIL	Inadequate
283	PASS	PASS	FAIL	PASS	PASS	Inadequate
284	PASS	PASS	PASS	FAIL	FAIL	Inadequate
285	PASS	PASS	PASS	PASS	PASS	Adequate
286	PASS	PASS	PASS	PASS	PASS	Adequate

287	PASS	PASS	FAIL	PASS	PASS	Inadequate
288	PASS	PASS	PASS	FAIL	FAIL	Inadequate
289	FAIL	PASS	FAIL	PASS	FAIL	Inadequate
290	PASS	PASS	PASS	PASS	PASS	Adequate
291	FAIL	PASS	FAIL	PASS	FAIL	Inadequate
292	FAIL	PASS	FAIL	FAIL	PASS	Inadequate
293	PASS	PASS	FAIL	PASS	PASS	Inadequate
294	PASS	PASS	PASS	FAIL	PASS	Inadequate
295	PASS	PASS	FAIL	FAIL	FAIL	Inadequate
296	PASS	PASS	FAIL	PASS	PASS	Inadequate
297	PASS	PASS	PASS	FAIL	PASS	Inadequate
298	PASS	PASS	FAIL	FAIL	PASS	Inadequate
299	PASS	PASS	PASS	PASS	PASS	Adequate
300	PASS	PASS	FAIL	PASS	PASS	Inadequate
301	FAIL	PASS	FAIL	FAIL	PASS	Inadequate
302	PASS	PASS	FAIL	PASS	PASS	Inadequate
303	PASS	PASS	PASS	PASS	PASS	Adequate
304	PASS	PASS	FAIL	PASS	PASS	Inadequate
305	FAIL	PASS	PASS	PASS	PASS	Inadequate
306	PASS	PASS	PASS	PASS	PASS	Adequate
307	FAIL	PASS	PASS	PASS	PASS	Inadequate
308	PASS	PASS	PASS	PASS	PASS	Adequate
309	PASS	PASS	PASS	PASS	PASS	Adequate
310	PASS	PASS	PASS	PASS	PASS	Adequate
311	PASS	PASS	PASS	PASS	PASS	Adequate
312	PASS	PASS	PASS	PASS	PASS	Adequate
313	PASS	PASS	PASS	PASS	PASS	Adequate
314	PASS	PASS	PASS	PASS	PASS	Adequate
315	PASS	PASS	PASS	PASS	PASS	Adequate
316	PASS	PASS	PASS	PASS	PASS	Adequate
317	PASS	PASS	PASS	PASS	PASS	Adequate
318	PASS	PASS	PASS	PASS	FAIL	Inadequate
319	PASS	PASS	PASS	PASS	PASS	Adequate
320	PASS	PASS	PASS	PASS	FAIL	Inadequate
321	PASS	PASS	PASS	PASS	PASS	Adequate
322	PASS	PASS	FAIL	PASS	FAIL	Inadequate
323	PASS	PASS	FAIL	FAIL	PASS	Inadequate
324	PASS	PASS	FAIL	FAIL	PASS	Inadequate
325	FAIL	FAIL	FAIL	FAIL	PASS	Inadequate
326	PASS	PASS	PASS	PASS	PASS	Adequate
327	PASS	PASS	PASS	PASS	PASS	Adequate

328	PASS	PASS	PASS	PASS	PASS	Adequate
329	FAIL	PASS	FAIL	FAIL	FAIL	Inadequate
330	PASS	PASS	PASS	PASS	PASS	Adequate
331	PASS	PASS	PASS	PASS	PASS	Adequate
332	PASS	PASS	PASS	PASS	PASS	Adequate
333	PASS	PASS	PASS	PASS	PASS	Adequate
334	PASS	PASS	PASS	PASS	PASS	Adequate
335	PASS	PASS	PASS	PASS	PASS	Adequate
336	PASS	PASS	PASS	FAIL	PASS	Inadequate
337	PASS	PASS	PASS	PASS	PASS	Adequate
338	PASS	PASS	PASS	PASS	FAIL	Inadequate
339	PASS	PASS	PASS	PASS	PASS	Adequate
340	PASS	PASS	FAIL	PASS	FAIL	Inadequate
341	PASS	PASS	FAIL	PASS	PASS	Inadequate
342	PASS	PASS	PASS	PASS	FAIL	Inadequate
343	PASS	PASS	PASS	FAIL	PASS	Inadequate
344	FAIL	FAIL	FAIL	PASS	FAIL	Inadequate
345	FAIL	PASS	FAIL	FAIL	PASS	Inadequate
346	PASS	PASS	PASS	FAIL	PASS	Inadequate
347	PASS	PASS	PASS	PASS	PASS	Adequate
348	PASS	PASS	PASS	PASS	PASS	Adequate
349	FAIL	FAIL	FAIL	PASS	PASS	Inadequate
350	PASS	PASS	PASS	FAIL	PASS	Inadequate
351	FAIL	PASS	FAIL	FAIL	FAIL	Inadequate
352	PASS	PASS	PASS	PASS	PASS	Adequate
353	FAIL	PASS	FAIL	PASS	PASS	Inadequate
354	PASS	PASS	FAIL	FAIL	FAIL	Inadequate
355	PASS	PASS	PASS	PASS	PASS	Adequate
356	PASS	PASS	PASS	PASS	PASS	Adequate
357	PASS	PASS	PASS	PASS	FAIL	Inadequate
358	PASS	PASS	PASS	PASS	FAIL	Inadequate
359	PASS	PASS	PASS	PASS	PASS	Adequate
360	PASS	PASS	PASS	PASS	PASS	Adequate
361	PASS	PASS	FAIL	PASS	FAIL	Inadequate
362	FAIL	PASS	FAIL	FAIL	FAIL	Inadequate
363	PASS	PASS	PASS	PASS	PASS	Adequate
364	PASS	PASS	PASS	FAIL	PASS	Inadequate
365	PASS	PASS	FAIL	PASS	PASS	Inadequate
366	PASS	PASS	PASS	PASS	FAIL	Inadequate
367	PASS	PASS	PASS	PASS	PASS	Adequate
368	PASS	PASS	PASS	PASS	FAIL	Inadequate

369	PASS	PASS	PASS	PASS	PASS	Adequate
370	FAIL	PASS	FAIL	PASS	FAIL	Inadequate
371	PASS	FAIL	FAIL	PASS	FAIL	Inadequate
372	PASS	PASS	FAIL	PASS	PASS	Inadequate
373	PASS	PASS	PASS	PASS	PASS	Adequate
374	PASS	PASS	PASS	PASS	FAIL	Inadequate
375	PASS	PASS	PASS	PASS	PASS	Adequate
376	PASS	PASS	PASS	PASS	PASS	Adequate
377	PASS	PASS	PASS	PASS	FAIL	Inadequate
378	PASS	FAIL	FAIL	PASS	PASS	Inadequate
379	PASS	PASS	PASS	PASS	PASS	Adequate
380	PASS	PASS	FAIL	PASS	PASS	Inadequate
381	PASS	FAIL	PASS	PASS	FAIL	Inadequate
382	PASS	PASS	PASS	PASS	FAIL	Inadequate
383	PASS	PASS	PASS	PASS	FAIL	Inadequate
384	PASS	PASS	FAIL	PASS	PASS	Inadequate
385	PASS	PASS	PASS	PASS	PASS	Adequate
386	PASS	PASS	PASS	PASS	PASS	Adequate
387	FAIL	PASS	FAIL	FAIL	FAIL	Inadequate
388	FAIL	FAIL	FAIL	PASS	FAIL	Inadequate
389	PASS	PASS	PASS	PASS	FAIL	Inadequate
390	FAIL	PASS	FAIL	PASS	PASS	Inadequate
391	PASS	PASS	PASS	PASS	PASS	Adequate
392	FAIL	PASS	FAIL	PASS	FAIL	Inadequate
393	PASS	PASS	PASS	PASS	PASS	Adequate
394	FAIL	PASS	PASS	PASS	FAIL	Inadequate
395	PASS	PASS	PASS	PASS	PASS	Adequate
396	PASS	PASS	PASS	PASS	FAIL	Inadequate
397	PASS	PASS	PASS	PASS	PASS	Adequate
398	FAIL	PASS	FAIL	PASS	PASS	Inadequate
399	PASS	PASS	PASS	PASS	PASS	Adequate
400	PASS	PASS	PASS	PASS	PASS	Adequate
401	FAIL	PASS	PASS	PASS	PASS	Inadequate
402	FAIL	FAIL	PASS	PASS	PASS	Inadequate
403	PASS	PASS	PASS	PASS	PASS	Adequate
404	FAIL	PASS	FAIL	PASS	PASS	Inadequate
405	FAIL	PASS	FAIL	PASS	FAIL	Inadequate
406	FAIL	PASS	FAIL	PASS	PASS	Inadequate
407	PASS	PASS	PASS	PASS	PASS	Adequate
408	PASS	PASS	PASS	PASS	PASS	Adequate
409	PASS	FAIL	FAIL	PASS	PASS	Inadequate

410	PASS	PASS	PASS	PASS	PASS	Adequate
411	FAIL	PASS	FAIL	PASS	PASS	Inadequate
412	PASS	PASS	PASS	PASS	PASS	Adequate
413	PASS	PASS	FAIL	PASS	PASS	Inadequate
414	PASS	PASS	PASS	PASS	PASS	Adequate
415	FAIL	PASS	PASS	PASS	FAIL	Inadequate
416	PASS	PASS	PASS	PASS	FAIL	Inadequate
417	PASS	PASS	PASS	PASS	PASS	Adequate
418	PASS	PASS	PASS	PASS	PASS	Adequate
419	PASS	PASS	PASS	PASS	FAIL	Inadequate
420	PASS	PASS	PASS	PASS	FAIL	Inadequate
421	PASS	PASS	FAIL	PASS	PASS	Inadequate
422	PASS	PASS	PASS	PASS	PASS	Adequate
423	PASS	PASS	PASS	PASS	PASS	Adequate
424	PASS	FAIL	FAIL	FAIL	PASS	Inadequate
425	PASS	PASS	FAIL	PASS	PASS	Inadequate
426	PASS	PASS	FAIL	PASS	PASS	Inadequate
427	PASS	PASS	PASS	PASS	PASS	Adequate
428	PASS	PASS	PASS	PASS	FAIL	Inadequate
429	PASS	PASS	PASS	PASS	FAIL	Inadequate
430	FAIL	FAIL	FAIL	PASS	FAIL	Inadequate
431	FAIL	PASS	FAIL	FAIL	PASS	Inadequate
432	PASS	PASS	PASS	PASS	PASS	Adequate
433	FAIL	PASS	FAIL	PASS	FAIL	Inadequate
434	PASS	PASS	PASS	PASS	PASS	Adequate
435	PASS	PASS	PASS	PASS	PASS	Adequate
436	PASS	PASS	PASS	PASS	PASS	Adequate
437	PASS	PASS	PASS	PASS	FAIL	Inadequate
438	PASS	PASS	PASS	PASS	FAIL	Inadequate
439	PASS	PASS	PASS	PASS	PASS	Adequate
440	PASS	PASS	FAIL	FAIL	FAIL	Inadequate
441	PASS	PASS	PASS	PASS	PASS	Adequate
442	FAIL	FAIL	FAIL	PASS	PASS	Inadequate
443	PASS	PASS	PASS	FAIL	PASS	Inadequate
444	PASS	PASS	PASS	PASS	PASS	Adequate
445	PASS	PASS	PASS	PASS	PASS	Adequate
446	PASS	PASS	PASS	PASS	PASS	Adequate
447	PASS	PASS	PASS	FAIL	PASS	Inadequate
448	PASS	PASS	FAIL	PASS	PASS	Inadequate
449	PASS	PASS	FAIL	PASS	PASS	Inadequate
450	PASS	PASS	PASS	PASS	PASS	Adequate

451	PASS	PASS	PASS	FAIL	FAIL	Inadequate
452	PASS	PASS	PASS	PASS	PASS	Adequate
453	FAIL	PASS	FAIL	PASS	FAIL	Inadequate
454	PASS	PASS	FAIL	FAIL	PASS	Inadequate
455	FAIL	FAIL	FAIL	PASS	PASS	Inadequate
456	PASS	PASS	PASS	PASS	FAIL	Inadequate
457	PASS	PASS	PASS	PASS	PASS	Adequate
458	PASS	PASS	PASS	PASS	PASS	Adequate
459	PASS	PASS	PASS	PASS	FAIL	Inadequate
460	PASS	PASS	PASS	PASS	FAIL	Inadequate
461	**PASS**	PASS	PASS	**FAIL**	**PASS**	Inadequate
462	**PASS**	PASS	PASS	**PASS**	**PASS**	Adequate
463	FAIL	PASS	PASS	**FAIL**	**PASS**	Inadequate
464	**PASS**	PASS	PASS	**PASS**	**PASS**	Adequate
465	**PASS**	PASS	PASS	**PASS**	**PASS**	Adequate
466	**PASS**	PASS	PASS	**PASS**	**PASS**	Adequate
467	**PASS**	PASS	PASS	**PASS**	**PASS**	Adequate
468	**PASS**	PASS	PASS	**PASS**	**PASS**	Adequate
469	**PASS**	PASS	PASS	**FAIL**	**PASS**	Inadequate
470	**PASS**	PASS	PASS	**PASS**	**PASS**	Adequate
471	**PASS**	PASS	FAIL	**PASS**	**PASS**	Inadequate
472	**PASS**	PASS	FAIL	**FAIL**	**PASS**	Inadequate
473	**PASS**	PASS	FAIL	**FAIL**	**PASS**	Inadequate
474	**PASS**	PASS	FAIL	**FAIL**	**PASS**	Inadequate
475	**PASS**	PASS	FAIL	**PASS**	**PASS**	Inadequate
476	**PASS**	PASS	FAIL	**FAIL**	**PASS**	Inadequate
477	**PASS**	PASS	FAIL	**FAIL**	**PASS**	Inadequate
478	**PASS**	PASS	FAIL	**PASS**	**PASS**	Inadequate
479	**PASS**	PASS	FAIL	**FAIL**	**PASS**	Inadequate
480	**PASS**	PASS	FAIL	**PASS**	**PASS**	Inadequate
481	**PASS**	PASS	FAIL	**FAIL**	**PASS**	Inadequate
482	**PASS**	PASS	FAIL	**PASS**	**PASS**	Inadequate
483	**PASS**	PASS	FAIL	**FAIL**	**PASS**	Inadequate
484	**PASS**	PASS	FAIL	**FAIL**	**PASS**	Inadequate
485	**PASS**	PASS	FAIL	**PASS**	**PASS**	Inadequate
486	**PASS**	PASS	FAIL	**PASS**	**PASS**	Inadequate
487	**PASS**	PASS	PASS	**PASS**	**PASS**	Adequate
488	FAIL	PASS	PASS	**FAIL**	**PASS**	Inadequate
489	**PASS**	PASS	PASS	**PASS**	**PASS**	Adequate
490	**PASS**	PASS	PASS	**PASS**	**PASS**	Adequate
491	**PASS**	PASS	PASS	**PASS**	**PASS**	Adequate

492	**PASS**	PASS	PASS	**PASS**	**PASS**	Adequate
493	**PASS**	PASS	PASS	**PASS**	**PASS**	Adequate
494	**PASS**	PASS	PASS	**FAIL**	**PASS**	Inadequate
495	PASS	PASS	PASS	PASS	FAIL	Inadequate
496	PASS	PASS	FAIL	PASS	FAIL	Inadequate
497	PASS	PASS	FAIL	PASS	FAIL	Inadequate
498	PASS	PASS	FAIL	PASS	PASS	Inadequate
499	PASS	PASS	PASS	PASS	PASS	Adequate
500	PASS	PASS	FAIL	FAIL	FAIL	Inadequate
501	FAIL	PASS	FAIL	PASS	PASS	Inadequate
502	PASS	PASS	FAIL	PASS	FAIL	Inadequate
503	PASS	PASS	FAIL	PASS	FAIL	Inadequate
504	PASS	PASS	PASS	PASS	PASS	Adequate
505	PASS	PASS	FAIL	PASS	FAIL	Inadequate
506	PASS	PASS	FAIL	PASS	FAIL	Inadequate
507	PASS	PASS	PASS	PASS	PASS	Adequate
508	PASS	PASS	PASS	PASS	FAIL	Inadequate
509	PASS	PASS	FAIL	FAIL	FAIL	Inadequate
510	PASS	PASS	PASS	PASS	FAIL	Inadequate
511	PASS	PASS	PASS	PASS	PASS	Adequate
512	FAIL	PASS	FAIL	FAIL	FAIL	Inadequate
513	PASS	PASS	FAIL	PASS	FAIL	Inadequate
514	PASS	PASS	PASS	FAIL	PASS	Inadequate
515	FAIL	PASS	PASS	PASS	FAIL	Inadequate
516	PASS	PASS	FAIL	FAIL	FAIL	Inadequate
517	FAIL	PASS	PASS	FAIL	PASS	Inadequate
518	PASS	PASS	PASS	PASS	FAIL	Inadequate
519	PASS	PASS	PASS	PASS	PASS	Adequate
520	PASS	PASS	PASS	FAIL	PASS	Inadequate
521	PASS	PASS	PASS	PASS	PASS	Adequate
522	PASS	PASS	FAIL	PASS	PASS	Inadequate
523	FAIL	FAIL	FAIL	PASS	FAIL	Inadequate
524	FAIL	PASS	FAIL	PASS	FAIL	Inadequate
525	PASS	PASS	FAIL	FAIL	FAIL	Inadequate
526	PASS	PASS	FAIL	PASS	PASS	Inadequate
527	FAIL	FAIL	FAIL	FAIL	FAIL	Inadequate
528	PASS	PASS	FAIL	PASS	FAIL	Inadequate
529	PASS	PASS	FAIL	PASS	PASS	Inadequate
530	PASS	PASS	PASS	PASS	PASS	Adequate
531	PASS	PASS	FAIL	FAIL	FAIL	Inadequate
532	PASS	PASS	PASS	PASS	FAIL	Inadequate

533	PASS	PASS	PASS	PASS	FAIL	Inadequate
534	PASS	PASS	PASS	PASS	PASS	Adequate
535	PASS	PASS	FAIL	FAIL	FAIL	Inadequate
536	FAIL	PASS	PASS	PASS	FAIL	Inadequate
537	FAIL	PASS	PASS	PASS	FAIL	Inadequate
538	FAIL	PASS	PASS	PASS	FAIL	Inadequate
539	FAIL	PASS	PASS	PASS	FAIL	Inadequate
540	**PASS**	PASS	PASS	**FAIL**	**PASS**	Inadequate
541	**PASS**	PASS	PASS	**PASS**	**PASS**	Adequate
542	FAIL	PASS	PASS	**FAIL**	**PASS**	Inadequate
543	**PASS**	PASS	PASS	**PASS**	**PASS**	Adequate
544	**PASS**	PASS	PASS	**PASS**	**PASS**	Adequate
545	**PASS**	PASS	PASS	**PASS**	**PASS**	Adequate
546	**PASS**	PASS	FAIL	**FAIL**	**PASS**	Inadequate
547	**PASS**	PASS	FAIL	**FAIL**	**PASS**	Inadequate
548	**PASS**	PASS	FAIL	**PASS**	**PASS**	Inadequate
549	**PASS**	PASS	FAIL	**PASS**	**PASS**	Inadequate
550	FAIL	FAIL	FAIL	PASS	FAIL	Inadequate
551	PASS	PASS	FAIL	PASS	FAIL	Inadequate
552	PASS	PASS	PASS	PASS	FAIL	Inadequate
553	PASS	PASS	PASS	PASS	FAIL	Inadequate
554	PASS	PASS	FAIL	PASS	FAIL	Inadequate
555	PASS	PASS	PASS	PASS	FAIL	Inadequate
556	PASS	PASS	PASS	PASS	FAIL	Inadequate
557	FAIL	PASS	PASS	PASS	PASS	Inadequate
558	PASS	PASS	PASS	PASS	PASS	Adequate
559	PASS	PASS	PASS	PASS	PASS	Adequate
560	PASS	FAIL	FAIL	PASS	PASS	Inadequate
561	PASS	PASS	PASS	PASS	FAIL	Inadequate
562	PASS	PASS	PASS	PASS	FAIL	Inadequate
563	PASS	PASS	PASS	PASS	FAIL	Inadequate
564	PASS	PASS	PASS	PASS	FAIL	Inadequate
565	PASS	PASS	FAIL	FAIL	FAIL	Inadequate
566	PASS	PASS	PASS	PASS	PASS	Adequate
567	PASS	PASS	FAIL	PASS	FAIL	Inadequate
568	PASS	PASS	FAIL	PASS	PASS	Inadequate
569	FAIL	PASS	FAIL	PASS	FAIL	Inadequate
570	PASS	FAIL	PASS	PASS	FAIL	Inadequate
571	PASS	PASS	PASS	PASS	FAIL	Inadequate
572	PASS	PASS	PASS	PASS	FAIL	Inadequate
573	PASS	PASS	PASS	FAIL	FAIL	Inadequate

574	PASS	PASS	PASS	FAIL	FAIL	Inadequate
575	PASS	PASS	PASS	FAIL	FAIL	Inadequate
576	PASS	PASS	PASS	FAIL	FAIL	Inadequate
577	PASS	PASS	PASS	FAIL	FAIL	Inadequate
578	PASS	PASS	PASS	FAIL	FAIL	Inadequate
579	PASS	PASS	PASS	FAIL	FAIL	Inadequate
580	PASS	PASS	PASS	PASS	PASS	Adequate
581	PASS	PASS	PASS	PASS	FAIL	Inadequate
582	PASS	PASS	PASS	PASS	PASS	Adequate
583	PASS	PASS	PASS	PASS	PASS	Adequate
584	PASS	PASS	PASS	PASS	FAIL	Inadequate
585	PASS	FAIL	FAIL	PASS	PASS	Inadequate
586	PASS	PASS	PASS	PASS	PASS	Adequate
587	PASS	PASS	FAIL	PASS	PASS	Inadequate
588	PASS	FAIL	PASS	PASS	FAIL	Inadequate
589	PASS	PASS	PASS	PASS	FAIL	Inadequate
590	PASS	PASS	PASS	PASS	FAIL	Inadequate
591	PASS	PASS	FAIL	PASS	PASS	Inadequate
592	PASS	PASS	PASS	PASS	PASS	Adequate
593	PASS	PASS	PASS	PASS	PASS	Adequate
594	FAIL	PASS	FAIL	FAIL	FAIL	Inadequate
595	FAIL	FAIL	FAIL	PASS	FAIL	Inadequate
596	PASS	PASS	PASS	PASS	FAIL	Inadequate
597	FAIL	PASS	FAIL	PASS	PASS	Inadequate
598	PASS	PASS	PASS	PASS	PASS	Adequate
599	FAIL	PASS	FAIL	PASS	FAIL	Inadequate
600	PASS	PASS	PASS	PASS	PASS	Adequate
601	FAIL	PASS	PASS	PASS	FAIL	Inadequate
602	PASS	PASS	PASS	PASS	PASS	Adequate
603	PASS	PASS	PASS	PASS	FAIL	Inadequate
604	PASS	PASS	PASS	PASS	PASS	Adequate
605	FAIL	PASS	FAIL	PASS	PASS	Inadequate
606	PASS	PASS	PASS	PASS	PASS	Adequate
607	PASS	PASS	PASS	PASS	PASS	Adequate
608	**PASS**	PASS	PASS	**PASS**	**PASS**	Adequate
609	FAIL	PASS	PASS	**FAIL**	**PASS**	Inadequate
610	**PASS**	PASS	PASS	**PASS**	**PASS**	Adequate
611	**PASS**	PASS	PASS	**PASS**	**PASS**	Adequate
612	FAIL	PASS	PASS	FAIL	PASS	Inadequate
613	PASS	PASS	PASS	PASS	FAIL	Inadequate
614	PASS	PASS	PASS	PASS	PASS	Adequate

615	PASS	PASS	PASS	FAIL	PASS	Inadequate
616	PASS	PASS	PASS	PASS	PASS	Adequate
617	PASS	PASS	FAIL	PASS	PASS	Inadequate
618	FAIL	FAIL	FAIL	PASS	FAIL	Inadequate
619	FAIL	PASS	FAIL	PASS	FAIL	Inadequate
620	PASS	PASS	FAIL	FAIL	FAIL	Inadequate
621	PASS	PASS	FAIL	PASS	PASS	Inadequate
622	FAIL	FAIL	FAIL	FAIL	FAIL	Inadequate
623	PASS	PASS	FAIL	PASS	FAIL	Inadequate
624	PASS	PASS	FAIL	PASS	PASS	Inadequate
625	PASS	PASS	PASS	PASS	PASS	Adequate
626	PASS	PASS	FAIL	FAIL	FAIL	Inadequate
627	PASS	PASS	PASS	PASS	FAIL	Inadequate
628	PASS	PASS	PASS	PASS	FAIL	Inadequate
629	PASS	PASS	PASS	PASS	PASS	Adequate
630	PASS	PASS	FAIL	PASS	PASS	Inadequate
631	FAIL	FAIL	FAIL	PASS	FAIL	Inadequate
632	FAIL	PASS	FAIL	PASS	FAIL	Inadequate
633	PASS	PASS	FAIL	FAIL	FAIL	Inadequate
634	PASS	PASS	FAIL	PASS	PASS	Inadequate
635	FAIL	FAIL	FAIL	FAIL	FAIL	Inadequate
636	PASS	PASS	FAIL	PASS	FAIL	Inadequate
637	PASS	PASS	FAIL	PASS	PASS	Inadequate
638	PASS	PASS	PASS	PASS	PASS	Adequate
639	PASS	PASS	FAIL	FAIL	FAIL	Inadequate
640	PASS	PASS	PASS	PASS	FAIL	Inadequate
641	PASS	PASS	PASS	PASS	FAIL	Inadequate
642	PASS	PASS	PASS	PASS	PASS	Adequate
643	PASS	PASS	FAIL	FAIL	FAIL	Inadequate
644	FAIL	PASS	PASS	PASS	FAIL	Inadequate
645	FAIL	PASS	PASS	PASS	FAIL	Inadequate
646	FAIL	PASS	PASS	PASS	FAIL	Inadequate
647	FAIL	PASS	PASS	PASS	FAIL	Inadequate
648	**PASS**	PASS	PASS	**FAIL**	**PASS**	Inadequate
649	**PASS**	PASS	PASS	**PASS**	**PASS**	Adequate
650	FAIL	PASS	PASS	**FAIL**	**PASS**	Inadequate
651	**PASS**	PASS	PASS	**PASS**	**PASS**	Adequate
652	**PASS**	PASS	PASS	**PASS**	**PASS**	Adequate
653	**PASS**	PASS	PASS	**PASS**	**PASS**	Adequate
654	**PASS**	PASS	FAIL	**FAIL**	**PASS**	Inadequate
655	**PASS**	PASS	FAIL	**FAIL**	**PASS**	Inadequate

656	**PASS**	PASS	FAIL	**PASS**	**PASS**	Inadequate
657	**PASS**	PASS	FAIL	**PASS**	**PASS**	Inadequate
658	FAIL	FAIL	FAIL	PASS	FAIL	Inadequate
659	PASS	PASS	FAIL	PASS	FAIL	Inadequate
660	PASS	PASS	PASS	PASS	FAIL	Inadequate
661	PASS	PASS	PASS	PASS	FAIL	Inadequate
662	PASS	PASS	PASS	PASS	FAIL	Inadequate
663	PASS	PASS	FAIL	FAIL	FAIL	Inadequate
664	PASS	PASS	PASS	PASS	PASS	Adequate
665	PASS	PASS	FAIL	PASS	FAIL	Inadequate
666	PASS	PASS	FAIL	PASS	PASS	Inadequate
667	FAIL	PASS	FAIL	PASS	FAIL	Inadequate
668	PASS	FAIL	PASS	PASS	FAIL	Inadequate
669	PASS	PASS	PASS	PASS	FAIL	Inadequate
670	FAIL	PASS	FAIL	FAIL	FAIL	Inadequate
671	PASS	PASS	PASS	PASS	PASS	Adequate
672	FAIL	PASS	FAIL	PASS	PASS	Inadequate
673	PASS	PASS	FAIL	FAIL	FAIL	Inadequate
674	PASS	PASS	PASS	PASS	PASS	Adequate
675	PASS	PASS	PASS	PASS	PASS	Adequate
676	PASS	PASS	PASS	PASS	PASS	Adequate
677	PASS	PASS	PASS	FAIL	PASS	Inadequate
678	PASS	PASS	FAIL	PASS	PASS	Inadequate
679	PASS	PASS	PASS	PASS	FAIL	Inadequate
680	PASS	PASS	PASS	PASS	PASS	Adequate
681	PASS	PASS	PASS	PASS	FAIL	Inadequate
682	PASS	PASS	PASS	PASS	PASS	Adequate
683	FAIL	PASS	FAIL	PASS	FAIL	Inadequate
684	PASS	PASS	PASS	PASS	PASS	Adequate
685	FAIL	PASS	PASS	PASS	FAIL	Inadequate
686	PASS	PASS	PASS	PASS	PASS	Adequate
687	PASS	PASS	PASS	PASS	FAIL	Inadequate
688	PASS	PASS	PASS	PASS	PASS	Adequate
689	PASS	PASS	PASS	FAIL	PASS	Inadequate
690	FAIL	PASS	FAIL	FAIL	FAIL	Inadequate
691	PASS	PASS	PASS	PASS	PASS	Adequate
692	FAIL	PASS	FAIL	PASS	PASS	Inadequate
693	PASS	PASS	FAIL	FAIL	FAIL	Inadequate
694	PASS	PASS	PASS	PASS	PASS	Adequate
695	PASS	PASS	PASS	PASS	PASS	Adequate
696	PASS	PASS	PASS	PASS	FAIL	Inadequate

697	PASS	PASS	PASS	PASS	FAIL	Inadequate
698	PASS	PASS	PASS	PASS	PASS	Adequate
699	PASS	PASS	PASS	PASS	PASS	Adequate
700	PASS	PASS	FAIL	PASS	FAIL	Inadequate
701	FAIL	PASS	FAIL	FAIL	FAIL	Inadequate
702	PASS	PASS	PASS	PASS	PASS	Adequate
703	PASS	PASS	PASS	FAIL	PASS	Inadequate
704	PASS	PASS	FAIL	PASS	PASS	Inadequate
705	PASS	PASS	PASS	PASS	FAIL	Inadequate
706	PASS	PASS	PASS	PASS	PASS	Adequate
707	PASS	PASS	PASS	PASS	FAIL	Inadequate
708	PASS	PASS	PASS	PASS	PASS	Adequate
709	FAIL	PASS	FAIL	PASS	FAIL	Inadequate
710	PASS	FAIL	FAIL	PASS	FAIL	Inadequate
711	PASS	PASS	FAIL	PASS	PASS	Inadequate
712	PASS	FAIL	FAIL	PASS	PASS	Inadequate
713	PASS	PASS	PASS	PASS	PASS	Adequate
714	PASS	PASS	FAIL	PASS	PASS	Inadequate
715	PASS	FAIL	PASS	PASS	FAIL	Inadequate
716	PASS	PASS	PASS	PASS	FAIL	Inadequate
717	PASS	PASS	PASS	PASS	FAIL	Inadequate
718	PASS	PASS	FAIL	PASS	PASS	Inadequate
719	PASS	PASS	PASS	PASS	PASS	Adequate
720	PASS	PASS	PASS	PASS	PASS	Adequate
721	FAIL	PASS	FAIL	FAIL	FAIL	Inadequate
722	FAIL	FAIL	FAIL	PASS	FAIL	Inadequate
723	PASS	PASS	PASS	PASS	FAIL	Inadequate
724	FAIL	PASS	FAIL	PASS	PASS	Inadequate
725	PASS	PASS	PASS	PASS	PASS	Adequate
726	FAIL	PASS	FAIL	PASS	FAIL	Inadequate
727	PASS	PASS	PASS	PASS	PASS	Adequate
728	FAIL	PASS	PASS	PASS	FAIL	Inadequate
729	PASS	PASS	PASS	PASS	PASS	Adequate
730	PASS	PASS	PASS	PASS	FAIL	Inadequate
731	PASS	PASS	PASS	PASS	PASS	Adequate
732	FAIL	PASS	FAIL	PASS	PASS	Inadequate
733	PASS	PASS	PASS	PASS	PASS	Adequate
734	PASS	PASS	PASS	PASS	PASS	Adequate
735	FAIL	PASS	PASS	PASS	PASS	Inadequate
736	FAIL	FAIL	PASS	PASS	PASS	Inadequate
737	PASS	PASS	PASS	PASS	PASS	Adequate

738	FAIL	PASS	FAIL	PASS	PASS	Inadequate
739	FAIL	PASS	FAIL	PASS	FAIL	Inadequate
740	FAIL	PASS	FAIL	PASS	PASS	Inadequate
741	PASS	PASS	PASS	PASS	PASS	Adequate
742	PASS	PASS	PASS	PASS	PASS	Adequate
743	PASS	FAIL	FAIL	PASS	PASS	Inadequate
744	PASS	PASS	PASS	PASS	PASS	Adequate
745	FAIL	PASS	FAIL	PASS	PASS	Inadequate
746	PASS	PASS	PASS	PASS	PASS	Adequate
747	PASS	PASS	FAIL	PASS	PASS	Inadequate
748	PASS	PASS	PASS	PASS	PASS	Adequate
749	FAIL	PASS	PASS	PASS	FAIL	Inadequate
750	PASS	PASS	PASS	PASS	FAIL	Inadequate
751	PASS	PASS	PASS	PASS	PASS	Adequate
752	PASS	PASS	PASS	PASS	PASS	Adequate
753	PASS	PASS	PASS	PASS	FAIL	Inadequate
754	PASS	PASS	PASS	PASS	FAIL	Inadequate
755	**PASS**	PASS	PASS	**FAIL**	**PASS**	Inadequate
756	**PASS**	PASS	PASS	**PASS**	**PASS**	Adequate
757	FAIL	PASS	PASS	**FAIL**	**PASS**	Inadequate
758	**PASS**	PASS	PASS	**PASS**	**PASS**	Adequate
759	**PASS**	PASS	PASS	**PASS**	**PASS**	Adequate
760	**PASS**	PASS	PASS	**PASS**	**PASS**	Adequate
761	**PASS**	PASS	PASS	**PASS**	**PASS**	Adequate
762	**PASS**	PASS	PASS	**PASS**	**PASS**	Adequate
763	**PASS**	PASS	PASS	**FAIL**	**PASS**	Inadequate
764	**PASS**	PASS	PASS	**PASS**	**PASS**	Adequate
765	**PASS**	PASS	FAIL	**PASS**	**PASS**	Inadequate
766	**PASS**	PASS	FAIL	**FAIL**	**PASS**	Inadequate
767	FAIL	PASS	PASS	PASS	PASS	Inadequate
768	PASS	PASS	PASS	PASS	PASS	Adequate
769	PASS	PASS	PASS	PASS	PASS	Adequate
770	PASS	FAIL	FAIL	PASS	PASS	Inadequate
771	PASS	PASS	PASS	PASS	FAIL	Inadequate
772	PASS	PASS	PASS	PASS	FAIL	Inadequate
773	PASS	PASS	PASS	PASS	FAIL	Inadequate
774	PASS	PASS	PASS	PASS	FAIL	Inadequate
775	PASS	PASS	FAIL	FAIL	FAIL	Inadequate
776	PASS	PASS	PASS	PASS	PASS	Adequate
777	PASS	PASS	FAIL	PASS	FAIL	Inadequate
778	PASS	PASS	FAIL	PASS	PASS	Inadequate

779	FAIL	PASS	FAIL	PASS	FAIL	Inadequate
780	PASS	FAIL	PASS	PASS	FAIL	Inadequate
781	PASS	PASS	PASS	PASS	FAIL	Inadequate
782	PASS	PASS	PASS	PASS	FAIL	Inadequate
783	FAIL	PASS	FAIL	FAIL	FAIL	Inadequate
784	PASS	PASS	FAIL	PASS	FAIL	Inadequate
785	PASS	PASS	PASS	PASS	PASS	Adequate
786	PASS	PASS	PASS	PASS	FAIL	Inadequate
787	PASS	PASS	FAIL	PASS	PASS	Inadequate
788	PASS	PASS	PASS	PASS	FAIL	Inadequate
789	PASS	PASS	PASS	PASS	FAIL	Inadequate
790	PASS	PASS	PASS	PASS	PASS	Adequate
791	PASS	PASS	PASS	PASS	PASS	Adequate
792	PASS	PASS	PASS	PASS	PASS	Adequate
793	PASS	PASS	PASS	PASS	PASS	Adequate
794	PASS	PASS	PASS	PASS	PASS	Adequate
795	PASS	PASS	PASS	PASS	FAIL	Inadequate
796	PASS	PASS	PASS	PASS	PASS	Adequate
797	PASS	PASS	FAIL	PASS	FAIL	Inadequate
798	PASS	PASS	PASS	PASS	PASS	Adequate

Identify at least one mode of rabies transmission	Identify a potential reservoir species for rabies	Identify notable signs in rabid animals	Identify appropriate health seeking behaviour after an exposure has occurred	Identify the appropriate course of action to be taken with a suspect rabid animal after exposure
None	None	None	None	None
Dog bites	Dog	Salivation	Wash wound with antiseptic	Report to the nearest vet
Dog bite / licking wounds	Cat	Change in behaviour (e.g. a	Wash with water only	Report to the police
	Cat and Dog	Neurological signs	Go to the clinic/vet	Kill the dog
	Livestock	Combination of above	Go to the police	Other
	Dog and Livestock		Inform the owner	Any of the combinations
	Cat and Livestock		Any of the combinations	
	Other warm blooded mammals			
	Dog and other warm blooded mammals			
	Cat and Other			
	Livestock and Other			
	All warm blooded mammals			
	Dog, Cat and warm blooded mammals			
